# Nutritional Biomarkers as Predictors of Dysphonia Severity in Patients with Ischemic Stroke

**DOI:** 10.3390/nu15030652

**Published:** 2023-01-27

**Authors:** Ji Min Kim, Seung Don Yoo, Eo Jin Park

**Affiliations:** 1 Department of Rehabilitation Medicine, Kyung Hee University College of Medicine, Kyung Hee University Hospital at Gangdong, Seoul 05278, Republic of Korea; 2 Department of Medicine, AgeTech-Service Convergence Major, Kyung Hee University, Seoul 05278, Republic of Korea

**Keywords:** nutrition biomarkers, dysphonia severity index, maximum phonation time, ischemic stroke

## Abstract

Dysphonia and malnutrition are major problems in patients who have suffered an ischemic stroke. Tools to assess dysphonia severity include the dysphonia severity index (DSI) and maximum phonation time (MPT). This study aimed to investigate whether the nutritional biomarkers transferrin, albumin, and prealbumin could be predictors of dysphonia severity. A retrospective analysis was conducted between January 2018 and October 2022. A total of 180 patients who had suffered an ischemic stroke were included. Serum transferrin, albumin, and prealbumin levels were significantly correlated with DSI and MPT levels. In a multiple regression analysis, prealbumin and transferrin were significant predictors of DSI, whereas only prealbumin was a significant predictor of MPT. Serum transferrin, albumin, and prealbumin levels in patients who have suffered an ischemic stroke may correlate with dysphonia severity as assessed using DSI and MPT. These results may provide objective evidence that nutritional biomarkers affect dysphonia severity.

## 1. Introduction

Every year, there are more than 7.6 million new cases of an ischemic stroke reported all over the globe [1]. Ischemic strokes account for more than 62 percent of all strokes [1]. Ischemic strokes account for the deaths of 3.3 million individuals per year [1]. Several motor, sensory, cognitive, and communication disorders occur after ischemic stroke, and among them, dysphonia is an important problem that is easily overlooked in terms of the functional outcome of ischemic rehabilitation and the quality of life of patients. Dysphonia refers to changes in voice, such as hoarseness or pitch quality [2]. Dysphonia is common after an ischemic stroke and occurs in approximately 20% of patients [3]. Dysphonia causes communication difficulties and reduces the patient’s quality of life [4]. When listening to speakers who have dysphonia, listeners need more time to process the speech inputs they receive, and speakers also make more intelligibility mistakes [5]. Dysphonia also affects rehabilitation treatment compliance in patients who have suffered an ischemic stroke and may cause poor functional outcomes [6]. Dysphonia has also been reported to be associated with swallowing dysfunction and is known as a predictor of aspiration risk [7,8]. Aspiration pneumonia is a risk factor that increases mortality in patients who have suffered an ischemic stroke [9,10]. Therefore, predicting dysphonia in ischemic stroke survivors is important. The tools used to assess dysphonia severity include the maximum phonation time (MPT) and dysphonia severity index (DSI). The MPT evaluates the function of the vocal folds by holding /a/ vocalizations for as long as possible and measuring the duration [11]. The MPT evaluation reflects the severity of dysphonia by measuring how completely the vocal folds can be kept closed while maintaining a consistent pitch and volume during vocalization [12]. The function of the vocal folds is not only to produce sound but also to protect the airway from foreign objects. DSI was created to study phonetic functions of speech quantitatively [13]. This indicator consists of DSI developed to objectively and quantitatively evaluate voice function and consists of the jitter, lowest intensity (I-Low), highest fundamental frequency (F0-High), and MPT [13]. Jitter and shimmer are two types of frequency variation that are used to depict changes in the fundamental frequency [14]. Jitter refers to the variation or disturbance of fundamental frequency, while shimmer refers to the same disturbance but in relation to the intensity of voice emission or the amplitude of sound [15]. Jitter is brought on by a lack of control over vocal fold vibration and shimmer, in addition to a decrease in glottic resistance [16]. All of these factors are linked to the presence of noise during breathiness and emission [16]. Because of an irregularity in the coordination of the vocal folds, the heterogeneity of the vocal folds limits the higher vibration rates, resulting in a reduction in the F0-High [17]. Additionally, it raises the resistance of the vocal folds, which makes it necessary to apply a higher amount of driving pressure in order to commence and sustain vocalization [18]. As a consequence, there is an increase in the I-Low in dysphonia patients [13].

Malnutrition is common after an ischemic stroke, with a prevalence of up to 62% reported [19]. Malnutrition has been associated with increased mortality risk, longer hospital stays, and worse functional outcomes after an ischemic stroke [20,21]. Malnutrition is the outcome of a mismatch between nutritional needs and consumption. There is a complicated relationship between malnutrition and disease-related inflammation in many malnourished individuals [22]. Therefore, assessing an adequate nutritional status is difficult and important. Several biomarkers evaluate the nutritional status, including serum transferrin, albumin, and prealbumin. Albumin has been used as an indication of malnutrition in medically stable individuals for decades [23]. Low albumin levels are associated with a significant decrease in muscle mass in older adults [23]. Systemic inflammation diminishes albumin production, increases albumin breakdown, and enhances albumin transcapillary leakage [22]. However, it has been reported that albumin has a long half-life of approximately 20 days and lacks specificity for nutritional evaluation [24]. Serum albumin levels decreased during the synthesis of inflammatory cytokines or decreased synthesis due to hepatic dysfunction [25]. Serum albumin levels may also decrease due to renal loss in nephrotic syndrome and loss through the gastrointestinal tract in protein-losing enteropathy [25]. Prealbumin, also known as transthyretin, is a transport protein for a thyroid hormone created by the liver and is then partly degraded by the kidneys [4]. Less than 10 mg/dL of serum prealbumin is connected with malnutrition [26]. Compared with albumin, the advantage of prealbumin is that it has a short half-life of 2–3 days; thus, it can be a better indicator of rapid changes in malnutrition status. Moreover, intestinal protein losses had little influence on prealbumin [25]. Prealbumin may also be affected by acute inflammatory conditions, renal dysfunction, corticosteroid therapy, and liver dysfunction [27]. Transferrin is an iron transport protein and acute-phase reactant [28]. Prealbumin is produced in the choroid plexus and retina, and its association with functional outcomes and mortality after ischemic stroke has also been reported [29]. Transferrin has a half-life of approximately 10 days, shorter than albumin and longer than prealbumin, and has been used as a biomarker to evaluate malnutrition status [28]. It is also affected by iron status, liver illness, and inflammation. Similar to prealbumin, serum transferrin concentrations increase with renal dysfunction [30]. Several studies have shown that transferrin assays are useful for nutritional assessment [31]. During iron insufficiency, transferrin levels are high but lower during iron excess. During nutritional supplementation in severely ill patients, serum transferrin increased in parallel to prealbumin [32]. Serum transferrin concentrations are decreased in conditions of severe malnutrition [22].

The pathophysiology of dysphonia is characterized by irregularities in vocal fold oscillations owing to unequal muscle tone, which may be caused by hypertonicity, poor glottic closure during phonation, or alterations in vocal fold structure [33]. After the onset of an ischemic stroke, patients may present with reduced vocal fold movement and vocalization-related muscle weakness [34]. This may impede breathing, sound production, and vocal stability, thus interfering with the coordination of speech motions [33]. Malnutrition may exacerbate the loss of muscle mass and strength [35]. Skeletal muscle is susceptible to muscle protein dissociation in catabolic conditions, which often occur during malnutrition or severe sickness [36,37]. Malnutrition, especially in conjunction with physical inactivity, may thereby hasten the onset of muscle wasting, which can have devastating consequences [38,39]. Malnutrition is a major cause of sarcopenia which causes deterioration of laryngeal muscle movement [40]. Vocal fold muscles are also skeletal muscles and may be affected by malnutrition. Due to the comparable histological architecture of the tongue and thyroarytenoid muscles, sarcopenia may arise in the vocal fold muscles [41,42]. Weakness and wasting of the vocal fold muscles can cause changes in voice quality [41]. In addition, sarcopenia of the respiratory muscles may cause changes in voice quality and dysphonia due to a decrease in expiratory function [43]. However, there are no studies on the association between serum transferrin, albumin, and prealbumin and dysphonia severity in patients who have suffered an ischemic stroke. Therefore, this study aimed to investigate whether serum transferrin, albumin, and prealbumin could be predictors of dysphonia severity.

## 2. Methods

### 2.1. Participants

**Figure 1:**
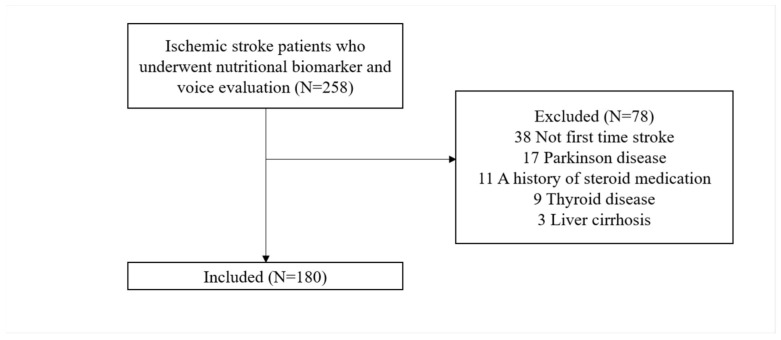
Flow chart of study participants enrollment.

A retrospective analysis was conducted between January 2018 and October 2022 on patients admitted an with ischemic stroke at the Kyung Hee University Hospital in Gangdong. Patients who were evaluated for serum transferrin, albumin, and prealbumin and who had a voice evaluation were recruited. Patients who had suffered an ischemic stroke for the first time and those who completed the evaluation within 1 month of onset were included. Patients with an acute inflammatory illness, a history of thyroid disease, nephrotic syndrome, liver disease, metabolic disease, or inflammatory bowel disease, and a history of steroid medication that might impact transferrin, albumin, and prealbumin levels were excluded. Patients with other brain diseases that could affect dysphonia severity, such as a brain tumor and traumatic brain injury, were also excluded. Patients with a cognitive impairment that made voice assessment difficult, aphasia, or tracheostomy were excluded. Patients with a history of chronic laryngitis, laryngopharyngeal reflux, polyps and nodules on the vocal fold, laryngeal cancer, Parkinson’s disease, and motor neuron diseases were excluded as these conditions could also cause dysphonia. Patients with a history of iatrogenic injury to the recurrent laryngeal or vagus nerves by thyroid and parathyroid surgery, carotid endarterectomy, or cardiothoracic procedures were also excluded (Figure 1). Dysphonia severity was compared between the normal value and low nutritional biomarker groups. A transferrin serum level of ≤200 mg/dL was designated as the low transferrin group [44], an albumin serum level of ≤3.5 g/dL was designated as the low albumin group [45], and a prealbumin serum level of ≤20 mg/dL was designated as the low prealbumin group [46]. The study was approved by the Institutional Review Board (IRB) of Kyung Hee University Hospital in Gangdong, Korea (IRB approval number: 2022-12-003).

### 2.2. Nutritional Biomarkers

The participants’ blood samples were collected, and nutritional biomarkers were measured. Serum levels of transferrin, albumin, and prealbumin were measured using AU5800 automatic chemistry analyzers (Beckman Coulter, Brea, CA, USA).

### 2.3. Maximum Phonation Time

Participants in the research vocalized the /a/ sound for as long as feasible after achieving maximal inspiration. The participants were advised to speak at a usual conversational volume level. The duration during which the vocalization was maintained with a steady volume and the audible sound was recorded. The individuals were instructed to sit in a relaxed posture, and the examination was administered three times. The maximum result of three measurements was recorded as MPT and measured in seconds (s) [12].

### 2.4. Dysphonia Severity Index

The sustained /a/ was evaluated for at least 3 s using the Model 3950 multidimensional voice software (Kay Pentax, Montvale, NJ, USA). Acoustic voice signals were evaluated for their F0-High, I-Low, jitter, and MPT. Individuals were advised to continue speaking the /a/ sound at a comfortable volume and pitch for 3 s to measure the jitter captured at 5 kHz [47,48]. To determine F0-High, /a/ was spoken at a standard pitch, which was elevated to the maximum achievable pitch [47]. After measuring I-Low at the participants’ usual pitch, they were told to gradually reduce the intensity for 5 s until they were whispering [47].

Positive DSI values indicate better vocal function, and negative DSI values indicate more severe dysphonia [48]. Afterward, the DSI was computed using the following formula: [48]

DSI = 0.13 × MPT + 0.0053 × F0-High − 0.26 × I-Low − 1.18 × jitter + 12.4 (1)

### 2.5. Statistical Analysis 

Using the Statistical Package for the Social Sciences version 20.0 for Windows (IBM Corp., Armonk, NY, USA), variables were statistically evaluated (IBM Corp., Armonk, NY, USA). The Kolmogorov–Smirnov and Levene tests were conducted to assess the normality of the data distribution and the homogeneity of the variance, respectively. Continuous variables were analyzed using the independent t-test, and categorical variables were analyzed using the chi-square test. Pearson’s correlation coefficient was used to analyze the relationship between serum transferrin, albumin, and prealbumin levels and MPT and DSI. Using multiple linear regression analysis with stepwise selection for sociodemographic characteristics, lifestyle characteristics, comorbidities, mean mini-mental state examination (MMSE) and modified Barthel index (MBI), the influence of serum transferrin, albumin, and prealbumin levels on the DSI and MPT was determined. In all statistical tests, a *p*-value less than 0.05 was deemed statistically significant.

## 3. Results

### 3.1. Demographic Characteristics of the Study Participants

The sociodemographic characteristics, lifestyle characteristics, brain lesion location, comorbidities, nutritional biomarkers, dysphonia severity are shown in Table 1. A total of 180 patients were included in this study, with a mean age of 61.46 ± 14.31 years and a sex distribution of 86 men and 94 women. Mean body mass index (BMI) was 22.41 ± 2.60 kg/m^2^. There were 61 patients with a history of smoking and 49 patients with a history of regular alcohol use. Brain lesion locations were the brainstem lesion in 103 patients and non-brain stem lesion in 77 patients, right hemisphere lesion in 73 patients, left hemisphere in 107 patients. Comorbidities were hypertension in 102 patients, arrhythmia in 70 patients, diabetes mellitus in 77 patients, dyslipidemia in 145 patients, heart failure in 42 patients, and coronary artery disease in 27 patients. The MMSE was 22.29 ± 3.11, the MBI was 40.04 ± 18.02, the serum albumin level was 2.97 ± 0.58 mg/dL, the serum prealbumin level was 19.59 ± 3.77 mg/dL, the serum transferrin level was 238.66 ± 99.66 mg/dL, DSI was −0.85 ± 2.20, and MPT was 11.58 ± 4.12 s. 

### 3.2. Comparison of Characteristics between Low Transferrin Group and Normal Value Group

Compared to the normal group, DSI (−1.66 ± 1.77, *p* = 0.002) and MPT (9.95 ± 4.21 s, *p* = 0.043) were statistically significantly lower in the low transferrin group. There were no statistically significant differences in age, sex, BMI, smoking history, regular alcohol use history, brain lesion location, MMSE score, or MBI. (Table 2).

### 3.3. Comparison of Characteristics between the Low Albumin Group and the Normal Value Group

Compared to the normal group, DSI (−1.51 ± 0.93, *p* = 0.013) and MPT (10.88 ± 3.55 s, *p* = 0.017) were statistically significantly lower in the low albumin group. The proportion of patients with a smoking history was significantly higher in the low albumin group (*p* = 0.047). There were no statistically significant differences in age, sex, BMI, alcohol use history, brain lesion location, MMSE score, or MBI. (Table 3).

### 3.4. Comparison of Characteristics between the Low Prealbumin Group and the Normal Value Group

Compared to the normal group, DSI (−1.61 ± 1.57, *p* < 0.001) and MPT (10.17 ± 3.75 s, *p* = 0.004) were statistically significantly lower in the low prealbumin group. There were no statistically significant differences in age, sex, BMI, smoking history, regular alcohol use history, brain lesion location, MMSE score, or MBI. (Table 4).

### 3.5. Association of Serum Transferrin, Albumin, and Prealbumin Level with DSI and MPT

Serum albumin level correlated significantly with DSI (r = 0.396, *p* = 0.04) and MPT (r = 0.386, *p* = 0.03). Serum prealbumin level was correlated significantly with DSI (R = 0.389, *p* < 0.001) and MPT (r = 0.388, *p* < 0.001). Serum transferrin level was significantly correlated with DSI (r = 0.416, *p* = 0.002) and MPT (r = 0.387, *p* = 0.008) (Table 5).

In a multiple linear regression analysis with stepwise selection, prealbumin (standardized β = 6.274, B = 3.663, *p* = 0.002) and transferrin (standardized β = 6.677, B = 0.148, *p* < 0.001) were significant predictors of DSI (adjusted R^2^ = 0.335). In addition, prealbumin (standardized β = 0.389, B = 0.425, *p* < 0.001) was a significant predictor of MPT (adjusted R^2^ = 0.147) (Table 6).

## 4. Discussion

To the best of our knowledge, this study is the first to evaluate the association between DSI and MPT and nutritional biomarkers measured in patients who had suffered an ischemic stroke. The serum levels of the nutritional biomarkers were divided into a low nutritional biomarker group and a normal value group as categorical variables, and a comparison was made between the two groups. As a result, MPT and DSI were significantly lower in the group with low nutritional biomarkers in all nutritional biomarkers. Serum transferrin, albumin, and prealbumin levels, which are used to determine nutritional status, were linked with MPT and DSI in this study. In addition, in a multiple linear regression analysis with stepwise selection, transferrin and prealbumin appeared to be significant predictors of DSI, and prealbumin appeared to be a significant predictor of MPT. These results are consistent with previous findings that prealbumin has a shorter half-life than albumin and is a sensitive indicator that reflects short-term impairment of energy intake and protein status in a more timely manner [49,50].

After an ischemic stroke, many patients develop dysphonia [3,4]. Patients with dysphonia complain of hoarseness, poor voice clarity, and discomfort when speaking. The strength and function of the laryngeal muscles that control the vocal folds are important in the vocalization process. The laryngeal muscle produces phonation, the sound energy created by the vocal fold vibration. However, the laryngeal muscle is usually paralyzed in patients who have suffered a stroke, resulting in reduced vocal fold movement and, therefore, vocalization problems [51,52]. Weakness of the laryngeal muscles is strongly associated with vocal fold instability [53,54]. Instability of the vocal folds may result in decreased phonetic function, as well as decreased coughing function, respiratory function, and swallowing function [55]. A study using laryngopharyngeal neuromuscular electrical stimulation showed improvement in dysphonia by increasing laryngeal elevation and improving excessive quaver through the strengthening of mylohyoid and thyrohyoid muscles [56]. Dysphonia is caused by weakness of the muscles controlling the vocal folds, and it is important to predict and evaluate the severity of dysphonia.

Since malnutrition is a major cause of sarcopenia and frailty syndrome, it is important to properly evaluate nutritional status. Transferrin, albumin, and prealbumin are widely used biomarkers that indicate nutritional status [23,57,58]. Malnutrition is an important causative factor for muscle loss [56]. Other studies have reported the role of transferrin in regulating neural regeneration or muscular atrophy [59]. There is also a report which found that low albumin levels are independently associated with a decrease in muscle strength [60]. Positive correlations exist between serum albumin levels, gait speed, and handgrip strength [61]. It has been found that higher serum albumin concentrations are associated with protective effects against skeletal muscle atrophy, decreased gait speed, and the occurrence of muscle wasting [62]. A study reported that prealbumin is more related to and better reflected in muscle mass loss than albumin [62]. The relationship between prealbumin levels and sarcopenia prevalence suggests that higher prealbumin levels may prevent older individuals from developing sarcopenia [62]. Sarcopenia may affect the muscles throughout the whole body, and it can also manifest itself in the form of respiratory sarcopenia and sarcopenic dysphagia [63,64]. The sarcopenia that might develop in these pharyngolaryngeal muscles can have an effect on the function of the voice. Since vocal fold muscles are skeletal muscles, malnutrition effects on the muscles may contribute to dysphonia severity.

MPT and DSI are objective tools for assessing dysphonia severity [48]. The results of this study found an association between MPT and DSI and nutritional biomarkers measured by prealbumin, albumin, and transferrin. This study suggests that nutritional biomarkers may be helpful as predictors of dysphonia severity in patients who had suffered an ischemic stroke. In addition, prealbumin was a significant predictor for both MPT and DSI. Although evaluating nutritional status as a single biomarker is not acceptable, it may be evidence that measuring prealbumin rather than measuring albumin alone may be helpful. It is required to review nutritional biomarkers that fit the demands of the evaluation since each nutritional biomarker has a varied half-life and a different index to reflect.

There were several limitations to this study. First, it was a retrospective cross-sectional study. Second, the proportion of patients with a smoking history was higher in the low albumin group than in the normal value group. Smoking history may have an impact on dysphonia and may have influenced the outcome [65]. Thus, for more reliable results, additional studies where smoking history variables are controlled are needed. Third, there may have been a selection bias by including only patients whose nutritional biomarkers were measured and who were evaluated for dysphonia. Fourth, there was a lack of information on whether malnutrition existed before an ischemic stroke occurred. Finally, although statistically significant results were obtained in the association analysis, the correlation coefficient value was low. Since it is somewhat difficult to conclude that there is a high correlation using only the results of this study, a large-scale prospective longitudinal study is needed in future.

## 5. Conclusions

In conclusion, serum transferrin, albumin, and prealbumin levels in patients with ischemic stroke may correlate with dysphonia severity as assessed using DSI and MPT. These results may provide objective evidence that nutritional biomarkers affect dysphonia severity.

## Figures and Tables

**Table 1:** Demographic characteristics of the study participants.

Characteristic	Value
Sociodemographic characteristics	
Age (years)	61.46 ± 14.31
Male	86 (47.80)
Female	94 (52.20)
Lifestyle characteristics	
BMI (kg/m^2^)	22.41 ± 2.60
Smoking	61 (33.90)
Regular alcohol use	49 (27.20)
Brain lesion location	
Brain stem	103 (57.20)
Non-brain stem	77 (42.80)
Right hemisphere	73 (40.60)
Left hemisphere	107 (59.40)
Comorbidities	
Hypertension	102 (56.70)
Arrhythmia	70 (38.90)
Diabetes mellitus	77 (42.80)
Dyslipidemia	145 (80.60)
Heart failure	42 (23.30)
Coronary artery disease	27 (15.00)
MMSE	22.29 ± 3.11
MBI	40.04 ± 18.02
Nutritional biomarkers	
Albumin (g/dL)	2.97 ± 0.58
Prealbumin (mg/dL)	19.59 ± 3.77
Transferrin (mg/dL)	238.66 ± 99.66
DSI	−0.85 ± 2.20
MPT (s)	11.58 ± 4.12

Values are presented as mean ± standard deviation or number (%). BMI, body mass index; MMSE, mini-mental state examination; MBI, modified Barthel index; DSI, dysphonia severity index; MPT, maximum phonation time.

**Table 2:** Comparison of characteristics between the low transferrin group and normal value group.

	Low Transferrin Group (*n*=75)	Normal Value Group (*n* = 105)	*p*-Value
Age (years)	61.59 ± 13.93	61.37 ± 14.64	0.921
Sex			0.960
Male	36 (48.00)	50 (47.60)	
Female	39 (52.00)	55 (52.40)	
BMI (kg/m^2^)	22.43 ± 2.66	22.39 ± 2.57	0.924
Smoking	24 (32.00)	37 (35.20)	0.651
Regular alcohol use	17 (22.70)	32 (30.50)	0.246
Brain lesion location			
Brain stem	46 (61.30)	57 (54.30)	0.346
Non-brain stem	29 (38.70)	48 (45.70)	
Right hemisphere	31 (41.30)	42 (40.00)	0.857
Left hemisphere	44 (58.70)	63 (60.00)	
MMSE	22.29 ± 3.20	22.29 ± 3.06	0.987
MBI	39.80 ± 17.32	40.22 ± 18.58	0.878
DSI	−1.66 ± 1.77	−0.27 ± 2.30	0.002 *
MPT (s)	9.95 ± 4.21	12.75 ± 3.65	0.043 *

Values are presented as mean ± standard deviation or number (%). BMI, body mass index; MMSE, Mini-Mental State Examination; MBI, modified Barthel index; DSI, dysphonia severity index; MPT, maximum phonation time. * *p* < 0.05.

**Table 3:** Comparison of characteristics between the low albumin group and the normal value group.

	Low Albumin Group (*n* = 131)	Normal Value Group (*n* = 49)	*p*-Value
Age (years)	61.58 ± 14.24	61.14 ± 14.63	0.856
Sex			0.843
Male	62 (47.30)	24 (49.00)	
Female	69 (52.70)	25 (51.00)	
BMI (kg/m^2^)	22.29 ± 2.56	22.74 ± 2.70	0.302
Smoking	50 (38.20)	11 (22.40)	0.047 *
Regular alcohol use	34 (26.00)	15 (30.60)	0.532
Brain lesion location			
Brain stem	76 (58.00)	27 (55.10)	0.725
Non-brain stem	55 (42.00)	22 (44.90)	
Right hemisphere	55 (42.00)	18 (36.70)	0.523
Left hemisphere	76 (58.00)	31 (63.30)	
MMSE	22.24 ± 3.16	22.43 ± 2.97	0.714
MBI	39.89 ± 17.99	40.45 ± 18.28	0.854
DSI	−1.51 ± 0.93	1.81 ± 2.17	0.013 *
MPT (s)	10.88 ± 3.55	13.44 ± 4.93	0.017 *

Values are presented as mean ± standard deviation or number (%). BMI, body mass index; MMSE, Mini-Mental State Examination; MBI, modified Barthel index; DSI, dysphonia severity index; MPT, maximum phonation time. * *p* < 0.05.

**Table 4:** Comparison of characteristics between the low prealbumin group and the normal value group.

	Low Prealbumin Group (*n* = 96)	Normal Value Group (*n* = 84)	*p*-Value
Age (years)	61.80 ± 13.62	61.07 ± 15.13	0.734
Sex			0.391
Male	43 (44.80)	43 (51.20)	
Female	53 (55.20)	41 (48.80)	
BMI (kg/m^2^)	22.35 ± 2.55	22.47 ± 2.68	0.756
Smoking	27 (28.10)	34 (40.50)	0.081
Regular alcohol use	24 (25.00)	25 (29.80)	0.474
Brain lesion location			
Brain stem	60 (62.50)	43 (51.20)	0.126
Non-brain stem	36 (37.50)	41 (48.80)	
Right hemisphere	40 (41.70)	33 (39.30)	0.746
Left hemisphere	56 (58.30)	51 (60.70)	
MMSE	22.18 ± 3.21	22.42 ± 3.00	0.608
MBI	38.81 ± 17.82	41.45 ± 18.24	0.328
DSI	−1.61 ± 1.57	0.02 ± 2.49	<0.001 **
MPT (s)	10.17 ± 3.75	13.20 ± 3.95	0.004 *

Values are presented as mean ± standard deviation or number (%). BMI, body mass index; MMSE, Mini-Mental State Examination; MBI, modified Barthel index; DSI, dysphonia severity index; MPT, maximum phonation time. * *p* < 0.05, ** *p* < 0.001.

**Table 5:** Correlation analysis of serum transferrin, albumin, and prealbumin level with DSI and MPT.

	Albumin	Prealbumin	Transferrin
DSI	*r* = 0.396	*r* = 0.389	*r* = 0.416
	*p* = 0.04 *	*p <* 0.001 **	*p* = 0.002 *
MPT	*r* = 0.386	*r* = 0.389	*r* = 0.387
	*p* = 0.03 *	*p* < 0.001 **	*p* = 0.008 *

DSI, dysphonia severity index; MPT, maximum phonation time. * *p* < 0.05, ** *p* < 0.001.

**Table 6:** Multiple linear regression analysis between nutritional markers, DSI, and MPT.

Dependent Variable	Independent Variable	Standardized β	B	95% CI	*p*-Value	Adjusted R^2^
DSI	Constant		35.639			0.335
	Transferrin	6.677	0.148	(0.106, 0.189)	<0.001 **	
	Prealbumin	6.274	3.663	(2.563, 4.762)	0.002*	
MPT	Constant		3.267			0.147
	Prealbumin	0.389	0.425	(0.276, 0.573)	<0.001 **	

Variables are based on their order of listing in the multiple regression analysis. DSI, dysphonia severity index; MPT, maximum phonation time; B, regression coefficient. * *p* < 0.05, ** *p* < 0.001.

## Data Availability

The datasets generated and/or analyzed during the current study are available from the corresponding author on reasonable request.
